# Dose-dependent exposure to indoxyl sulfate alters AHR signaling, sirtuins gene expression, oxidative DNA damage, and bone mineral status in rats

**DOI:** 10.1038/s41598-024-53164-3

**Published:** 2024-01-31

**Authors:** Malgorzata Karbowska, Krystyna Pawlak, Beata Sieklucka, Tomasz Domaniewski, Urszula Lebkowska, Radoslaw Zawadzki, Dariusz Pawlak

**Affiliations:** 1 Department of Pharmacodynamics, Medical University of Bialystok, Białystok, Poland; 2 Department of Monitored Pharmacotherapy, Medical University of Bialystok, Białystok, Poland; 3 Department of Radiology, Medical University of Bialystok, Białystok, Poland

**Keywords:** Physiology, Nephrology, Pathogenesis, Risk factors

## Abstract

Indoxyl sulfate (IS), an agonist of aryl hydrocarbon receptors (AhR), can accumulate in patients with chronic kidney disease, but its direct effect on bone is not clear. The present study investigated the effect of chronic exposure to low (100 mg/kg b.w.; 100 IS) and high (200 mg/kg b.w.; 200 IS) dose of IS on bone AhR pathway, sirtuins (SIRTs) expression, oxidative DNA damage and bone mineral status in Wistar rats. The accumulation of IS was observed only in trabecular bone tissue in both doses. The differences were observed in the bone parameters, depending on the applied IS dose. The exposure to 100 IS increased AhR repressor (AhRR)-CYP1A2 gene expression, which was associated with SIRT-1, SIRT-3 and SIRT-7 expression. At the low dose group, the oxidative DNA damage marker was unchanged in the bone samples, and it was inversely related to the abovementioned SIRTs expression. In contrast, the exposure to 200 IS reduced the expression of AhRR, CYP1A, SIRT-3 and SIRT-7 genes compared to 100 IS. The level of oxidative DNA damage was higher in trabecular bone in 200 IS group. Femoral bone mineral density was decreased, and inverse relations were noticed between the level of trabecular oxidative DNA damage and parameters of bone mineral status. In conclusion, IS modulates AhR-depending signaling affecting SIRTs expression, oxidative DNA damage and bone mineral status in a dose dependent manner.

## Introduction

Chronic kidney disease (CKD) is a serious global health problem that is associated with abnormalities in bone metabolism and bone mineralization called Chronic Kidney Disease Mineral and Bone Disorder (CKD-MBD). In the course of CKD-MBD patients suffer from increased number of fractures, osteoporosis and vascular calcification, which in consequence lead to the excess mortality among those patients^1^.

Indoxyl sulfate (IS) is one of uremic toxins that is retained during CKD. Recent reports demonstrate its harmful effect on*, inter alia*, cardiovascular and central nervous systems. It also exhibits prooxidative and pro-inflammatory properties, and has negative influence on bone formation and bone resorption^2^. In vitro studies indicate that IS modulates the differentiation and activation of osteoblasts and osteoclasts^3^, however IS direct impact on bone and mineral disorders still remains unclear.

IS is also an agonist of aryl hydrocarbon receptors (AhR), a ligand-activated transcription factor that belongs to the Per-Arnt-Sim superfamily of proteins^4^. The activation of AhR exerted the negative effect on the geometry and biomechanical properties of bone^5,6^. Moreover, it was demonstrated that activation of this receptor leads to the inhibition of osteoblast proliferation and differentiation. After a ligand is bound to AhR, the receptor translocates into the nucleus and forms an active heterodimer with aryl hydrocarbon receptor nuclear translocator (ARNT), which binds to xenobiotic responsive elements (XRE). This leads to induction of target genes, such as cytochrome P450 family 1 subfamily A (CYP1A), in order to promote the elimination of the AhR ligand. Simultaneously, the expression of AhR gene is activated, resulting in the renewal of AhR protein within the cell^7^. AhR repressor (AhRR) is another of the key target genes activated in the AhR pathway. AhRR is similar to the AhR but it cannot bind ligands, thus it suppresses AhR activity by creating AhRR-ARNT complex^8^. This negative regulatory loop and the proteosomal degradation of the AhR protect cells from the consequences of excessive stimulation by AhR agonist, ensuring a temporal control of the signaling^7,9^.

Sirtuins (SIRTs) are members of class III type nicotinamide adenine dinucleotide dependent histone deacetylases that are engaged in various processes like antioxidant defense, DNA damage repair, cellular homeostasis or longevity. Recent studies also show their crucial role in skeletal development and maintaining bone homeostasis^10^. Sirtuin 1 (SIRT-1) is the best studied SIRT, which is connected with differentiation of skeletal stem cells into osteoblasts. Decreased expression and activity of SIRT-1 lead to bone mass reduction^11^. Moreover, SIRT-1 is a negative regulator of Sost gene, which encodes sclerostin—an inhibitor of bone formation^12^. In turn, SIRT-2 exerts anti-inflammatory effect and reduces reactive oxygen species (ROS) level^13^. There is a little knowledge about its direct impact on bone. In vitro study demonstrated that inhibition of SIRT-2 by AGK2 compound suppressed the differentiation of bone marrow-derived mononuclear cells into osteoclasts^14^. Another SIRT—SIRT-3 reduces oxidative stress damage and weakens cell senescence^15^. Animal studies showed that SIRT-3 deficiency resulted in osteopenia and osteoblast dysfunction, which was connected with mitochondrial stress, as well as decreased bone mass due to an increased number of osteoclasts^16^. SIRT-3 negatively regulates osteoclastogenesis induced by receptor activator of nuclear factor κB ligand (RANKL)^17^. In addition, SIRT-7 plays crucial role in bone formation by regulating acylation of osterix, transcription factor necessary for the osteoblast differentiation and bone mineralization^18^. Available studies demonstrated that deficiency of SIRT-7 is associated with low bone mass^19^.

Studies conducted by other authors in various experimental models suggested the existence of the interrelationships between AhR and the SIRTs system^20–23^. We also noticed that chronic exposure to IS promotes arterial thrombosis and simultaneously decreased aortic contents of SIRT-1 and SIRT-3^24^, which leads us to believe that there is a link between AhR pathway activation and SIRTs at the tissue level.

Despite increasingly advanced diagnostic techniques, the pathophysiology of CKD-MBD raises many doubts due to its complexity. The purpose of the present study was to determine the direct effect of IS, one of the most potent protein-bound uremic toxins, on bone. Current knowledge of IS impact on bones comes from CKD patients and animals’ studies, which does not allow to observe IS effect solely due to the presence of other toxins creating „uremic milieu” that can exert similar effects on bones. Therefore, we planned a unique experimental model to evaluate IS level in the bone after chronic administration IS to control group, and its sole impact on AhR pathway, SIRTs expression, oxidative DNA damage and bone mineral status. The uptake of IS is closely associated with organic anion transporters (OATs), specifically isoforms OAT-1 and OAT-3. These transporters are responsible for facilitating the movement of IS in various tissues, such as renal proximal tubules and osteoblasts^2^. Blocking OATs with certain compounds has the potential to retain more IS in the circulatory system, thus helping to remove IS^2^. Accordingly, we also decided to evaluate OAT-1 and OAT-3 gene expression in our model.

## Methods

### Animals and experimental design

Details about the animals’ characteristics, experimental design and applied procedures were described previously^24^. To investigate the influence on low and high doses of IS, we divided 48 male Wistar Crl:WI (cmdb) rats (7 weeks old) into two groups—100 IS group receiving IS in the drinking water in the dose of 100 mg/kg of b.w./day (n = 16) and 200 IS group receiving IS in the drinking water in the dose of 200 mg/kg of b.w./day (n = 16). The control group (CON) received pure water (n = 16). The exposure time lasted for 28 days. All researchers obtained ethical clearance for conducting experiments on animals from Polish Laboratory Animal Science Association (PolLASA). All procedures involving animals were approved by the Institutional Local Ethics Committee of the University of Warmia and Mazury in Olsztyn (Agreement No. 124/2015 and No. 31/2017/WNP), and conducted by ARRIVE guidelines, and directive 2010/63/EU of the European Parliament and of the Council on the protection of animals used for scientific purposes.

### Preparation of bone tissue homogenates

The bone tissue was taken from the distal femoral epiphysis (trabecular bone) and femoral diaphysis (cortical bone) after 28 days of exposure to IS. The procedure of the preparation of 10% homogenates from bone tissue has been described in detail previously^25^.

### Determination of IS in bone homogenates

Concentrations of free form of IS in the bone tissue homogenates were evaluated using high-performance liquid chromatography (HPLC) with fluorescence detection according to our modification^24^ of the methods previously described by Al’Zhabi et al.^26^. Determination of IS was carried out at temperature of 24 °C. The chromatographic equipment was an Agilent 1200 series LC-system (Agilent Technologies, Boblingen, Germany) composed of G1322A degasser, G1311A quaternary pump, G1329A autosampler and G1330B thermostat for autosampler, HP1046A fluorescence detector (FLD). Deproteinated samples were prepared by adding 0.4 mL acetonitrile containing the methyl paraben (1 mg/mL) as internal standard into the 0.1 mL homogenate. The samples were vortexed, kept at 4 °C for 1 min, and then centrifuged for 30 min 14,000 g at 4 °C. Further, 1 µL of the supernatant was injected into high performance liquid chromatography (HPLC) system for analysis. The prepared samples were separated on column Phenomenex PEPTIDE 3.6mmXB-C18 4.6 × 250 mm (Phenomenex, Torrance, CA, USA). The column effluent was monitored by using programmable FLD. The optimized conditions were determined by recording fluorescence spectra with a stop-flow technique. Excitation and emission wavelengths were set at 280/375 nm. The output of the detector was connected to a single instrument LC ChemStation. The mobile phase was composed of acetate buffer (pH 4.5) containing 90% of acetonitrile and it was pumped at a flow-rate of 0.8 mL/min.

### Oxidative status assay in bone homogenates

The levels of 8-hydroxy-2'-deoxyguanosine (8-OHdG) were measured in the femur homogenates with DNA damage ELISA kit from Enzo Life Sciences (ELS) AG, Lausen, Switzerland, according to the manufacturer's instructions.

### Quantitative real-time polymerase chain reaction (qRT-PCR) assay

Total RNA was isolated from femoral bone using Thermo Scientific GeneJET RNA Purification Kit (Thermo Scientific, Vilnius, Lithuania) and a quantitative real-time polymerase chain reaction assay was performed as previously described^25^. Primers were designed using PRIMER-BLAST (http://www.ncbi.nlm.nih.gov/tools/primerblast) software. In the present study, the expression of sirtuins and AhR pathway genes were determined. The primer sequences used (50—30 forward, reverse) are included in the Table 1. All results were normalized to the endogenous reference glyceraldehyde 3—phosphate dehydrogenase (GADPH). The comparative cycle threshold method was used for relative quantification of gene expression.

**Table 1:** Primer sequences used in the Quantitative Real-time Polymerase Chain Reaction (qRT-PCR) assay.

50—30 forward, reverse primer sequence	Gene
ACAGTTTTCCGGCTTCTTGC, GTTCGCGTCCTTCTTCATCC	AhR
TTCATTTGTCGTGTCCGCTG, ATGCAAAACAAGGAGAGCCG	AhRR
GCCACAGGAGCTCTTAGGAA, GCTCGGAATCGGAACATGAC	ARNT
AGTTCAGTCCTTCCTCACAGC, TGAAGGCTGGGAATCCATACA	CYP1A1
GAGATGCTCAACCTCGTGAA, TTGGGCTCTGTCACAAGTAG	CYP1A2
CTCGCCTTGCTGTGGACTTC, TGTGACACAGAGATGGCTGG	SIRT-1
CCCCACTGTAACCACGTCTG, CTCCCTCAGTGTCCGAGTCT	SIRT-2
TGTGGGGTCCGGGAGTATTA, TGTGTCCTCCACATCCAAAGC	SIRT-3
GGCCTGGAGATCCCTGTCTA, ACTGTGGCTACCTTCTTCGC	SIRT-7
ACTGCATCTTCCTGTACACCG, GAGGCATGGAGGGGTAGAAC	OAT-1
ACCCAAATGGGAAGCCTGAG, GGTCCTCCAACCAGTATGCC	OAT-3
AAGATGGTGAAGGTCGGTGT, AGGTCAATGAAGGGGTCGTT	GADPH

AhR—aryl hydrocarbon receptor; AhRR—aryl hydrocarbon receptor repressor; ARNT—aryl hydrocarbon receptor nuclear translocator; GADPH—glyceraldehyde 3—phosphate dehydrogenase; OAT-1—organic anion transporter 1; OAT-3—organic anion transporter 3; SIRT-1—sirtuin 1; SIRT-2—sirtuin 2; SIRT-3—sirtuin 3; SIRT-7—sirtuin 7.

### Bone mineral status determination

Densitometry analysis of left femurs was performed using dual-energy x-ray absorptiometry (DXA) scans (Horizon QDR Series X-ray Bone Densitometer, Hologic Inc., Bedford, MA, USA), and analyzed with the specific small animal software. For each scanned femur the results of the bone mineral area (BMA, cm^2^), bone mineral content (BMC, mg), and areal bone mineral density (BMD, mg/cm^2^) were obtained. The variation coefficient of BMC and BMD measurements were less than 4%.

### Statistical analysis

Shapiro–Wilk's test of normality was used for data distribution analysis. The normally distributed data were presented as mean ± SD. The non-Gaussian data were expressed as median (interquartile range; IQR). Comparison between parametric data was made by one-way analysis of variance (ANOVA), and significant differences between the groups were assessed using Tukey’s post-hoc test at p < 0.05. The Kruskal–Wallis with Dunn's test was used for nonparametric data at p < 0.05. The correlations were calculated by Spearman's rank correlation analysis. p-value < 0.05 was considered statistically significant. Graphic design presentation of results was prepared using GraphPad Prism 6 (GraphPad Software, La Jolla, CA, USA) or Statistica ver.10 computer software (StatSoft, Tulsa, OK, USA).

## Results

### The concentration of IS in bones regions and the expression of IS transporters in bone

**Figure 1:**
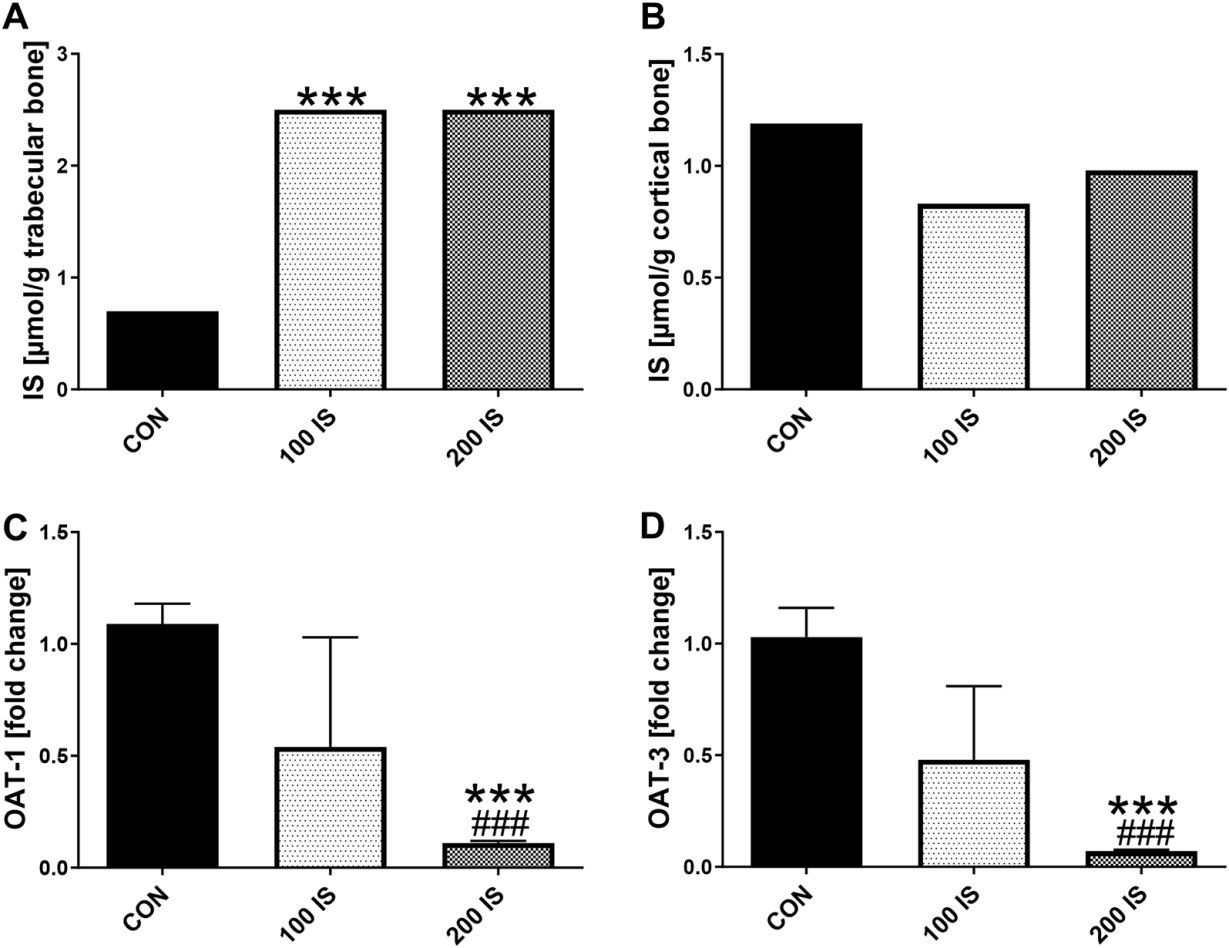
IS levels and the expression of IS transporters (OATs) in bone tissue of rats after chronic exposure to IS. Data are shown as the mean ± SD (**C**,**D**) or median (**A**,**B**) depending on their distribution. CON—control group; IS—indoxyl sulfate; 100 IS—group receiving indoxyl sulfate in the dose of 100 mg/kg b.w./day; 200 IS—group receiving indoxyl sulfate in the dose of 200 mg/kg b.w./day; OAT-1—organic anion transporter 1; OAT-3—organic anion transporter 3. *** p < 0.001 compared to control; ### p < 0.001 compared to 100-IS group.

As shown in Fig. 1A, chronic exposure to IS in both doses resulted in increased IS levels in trabecular regions of bones compared to CON group (p < 0.001). The median concentrations of IS were 2.5 µmol/g (IQR 1.36–9.12) in 100 IS group and 2.5 µmol/g (IQR 1.71–9.56) in 200 IS group. There were no differences in IS levels in cortical regions of bones (Fig. 1B).The only downward trend was observed in 100 IS group (p = 0.063; Fig. 1B). We did not notice any correlation between plasma IS levels and bone IS concentrations.

The expression of organic anion transporters OAT-1 and OAT-3 genes, which represented the transporters with high affinity to IS^27^ was non-significantly decreased at a lower dose of IS, and was strongly inhibited at a dose of 200 mg IS/kg b.w. compared to CON and 100 IS groups (p < 0.001, Fig. 1C,D). Despite such strong inhibition of OATs expression in 200 IS group, there was no direct association between plasma IS level and the expression of OAT-1 (R = − 0.354, NS) and OAT-3 (R = − 0.293, NS) genes.

### The impact of IS on AhR pathway genes expression in bone

**Figure 2:**
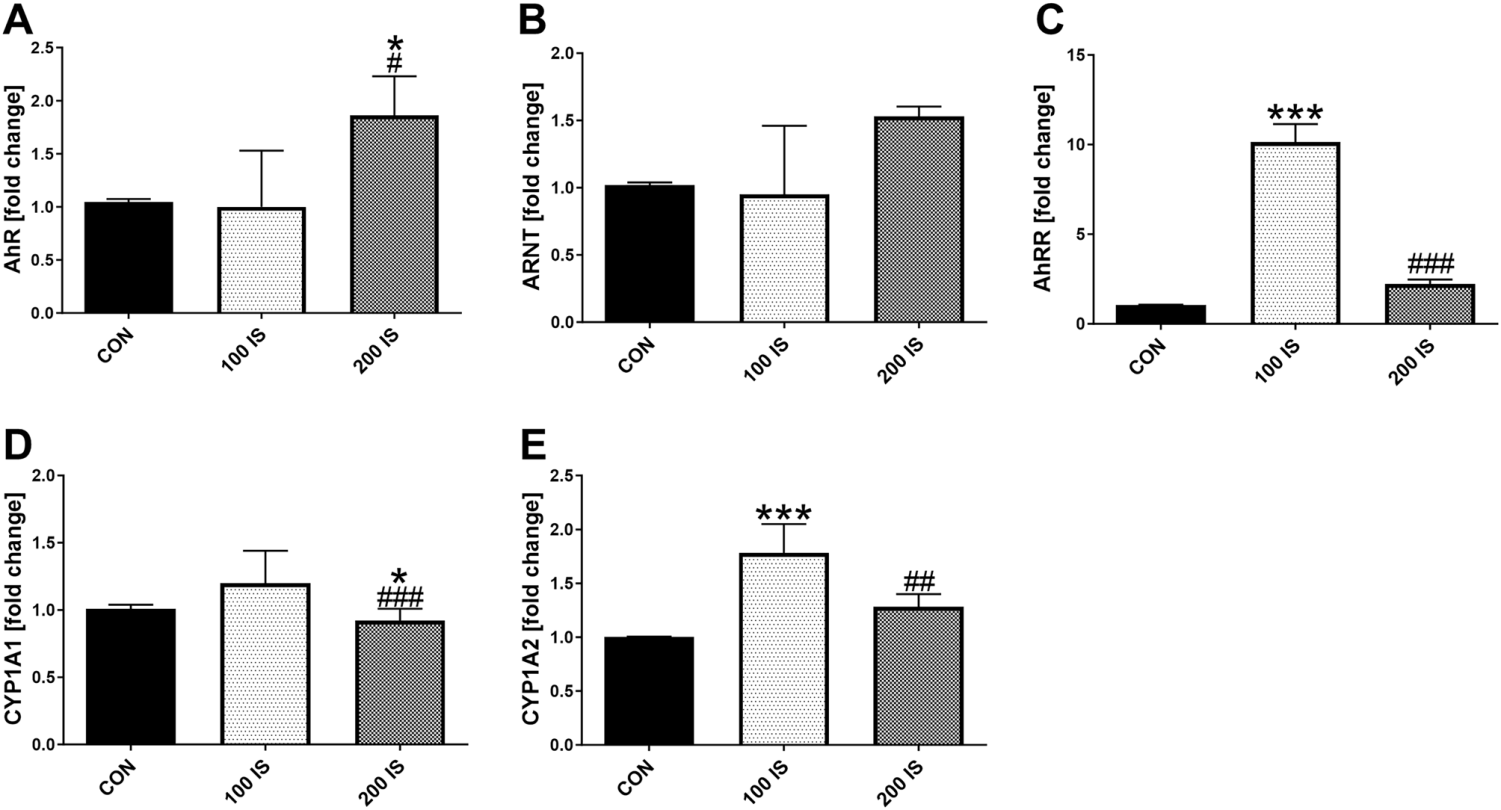
The expression of AhR pathway genes in bone tissue of rats after chronic exposure to IS. Data are shown as the mean ± SD. AhR—aryl hydrocarbon receptor; ; AhRR—aryl hydrocarbon receptor repressor; ARNT—aryl hydrocarbon receptor nuclear translocator; CON—control group; IS—indoxyl sulfate; 100 IS—group receiving indoxyl sulfate in the dose of 100 mg/kg b.w./day; 200 IS—group receiving indoxyl sulfate in the dose of 200 mg/kg b.w./day. * p < 0.05 compared to control; *** p < 0.001 compared to control; # p < 0.05 compared to 100-IS group; ## p < 0.01 compared to 100 IS group; ### p < 0.001 compared to 100 IS group.

The different effects of IS on the expression of the AhR pathway genes in bone were noticed in experimental groups (Fig. 2). In 100 IS group, the mRNA level of AhRR was tenfold higher, and CYP1A2 was 1.8-fold higher compared to CON group. The administration of high doses of IS resulted in increased expression of AhR compared to CON (p = 0.029) and 100 IS (p = 0.043) groups.. While, the expression of AhR repressor (AhRR), CYP1A1 and CYP1A2—the key target genes activated by the genomic AhR pathway was significantly inhibited in 200 IS group in comparison with 100 IS group (p < 0.001, p < 0.001 and p = 0.005, respectively) .

The analysis of associations existed between AhR pathway genes revealed inverse relations between CYP1A1 and the expression of OAT-3, ARNT and AhRR in the group exposed to lower IS dose. In turn, the strong positive correlation was observed between CYP1A2 and AhRR expression in this group (Table 2, left side). In the 200 IS group, a significant positive association was present between OAT-1 and analyzed genes that belong to the AhR pathway, except for CYP1A1. Moreover, all these genes were positively related each to others (Table 2, right side).

**Table 2:** The associations between genes involved in AhR-dependent pathway in bone of healthy rats after chronic exposure to indoxyl sulphate.

	100 IS						200 IS				
	OAT-1	AhR	ARNT	AhRR	CYP1A1		OAT-1	AhR	ARNT	AhRR	CYP1A2
OAT-1		R = 0.159 NS	R = − 0.049 NS	*R* = *0.527* *p* = *0.064*	R = − 0.445 NS	OAT-1		R = 0.421 NS	**R = 0.729** **p = 0.001**	R = 0.421 NS	**R = 0.706** **p = 0.002**
OAT-3	**R = 0.825** **p < 0.001**	R = 0.462 NS	R = 0.093 NS	R = 0.148 NS	**R = **− **0.588** **p = 0.034**	OAT-3	R = 0.446 NS	R = − 0.032 NS	**R = 0.538** **p = 0.047**	R = 0.385 NS	R = 0.068 NS
AhR	R = 0.159 NS		R = 0.093 NS	R = − 0.171 NS	R = 0.030 NS	AhR	**R = 0.625** **p = 0.013**		R = 0.332 NS	**R = 0.621** **p = 0.013**	**R = 0.604** **p = 0.017**
ARNT	R = − 0.049 NS	R = 0.093 NS		R = 0.197 NS	*R* = − *0.438* *p* = *0.089*	ARNT	**R = 0.729** **p = 0.001**	R = 0.332 NS		R = 0.288 NS	R = 0.306 NS
AhRR	*R* = *0.527* *p* = *0.064*	R = − 0.171 NS	R = 0.197 NS		R = − 0.359 NS	AhRR	R = 0.421 NS	**R = 0.621** **p = 0.013**	R = 0.288 NS		R = 0.403 NS
CYP1A2	R = 0.319 NS	R = − 0.124 NS	R = − 0.271 NS	**R = 0.694** **p = 0.003**	R = − 0.144 NS	CYP1A2	**R = 0.706** **p = 0.002**	**R = 0.604** **p = 0.017**	R = 0.306 NS	R = 0.403 NS	

AhR—aryl hydrocarbon receptor; AhRR—aryl hydrocarbon receptor repressor; ARNT—aryl hydrocarbon receptor nuclear translocator; 100 IS—group receiving indoxyl sulphate in the dose of 100 mg/kg b.w./day; 200 IS—group receiving indoxyl sulphate in the dose of 200 mg/kg b.w./day; OAT-1—organic anion transporter 1; OAT-3—organic anion transporter 3.

Bold values mean that correlation was statistically significant. Tendencies  are in italic.

Interestingly, we noticed that plasma IS levels were positively related to bone CYP1A1 expression in CON group (R = 0.601, p = 0.017) and 200 IS group (R = 0.538, p = 0.047), whereas trabecular IS concentrations were significantly associated with CYP1A2 gene expression only in CON group (R = 0.535, p = 0.033).

### The effect of chronic exposure to exogenous IS on SIRTs expression in bone

**Figure 3:**
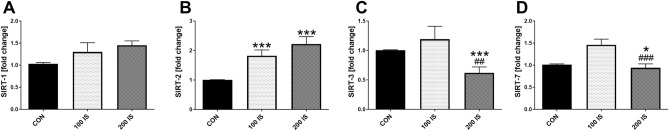
The bone expression of SIRTs genes (**A**–**D**). Data are shown as the mean ± SD. CON—control group; 100 IS—group receiving indoxyl sulfate in the dose of 100 mg/kg b.w./day; 200 IS—group receiving indoxyl sulfate in the dose of 200 mg/kg b.w./day; SIRT-1—sirtuin 1; SIRT-2—sirtuin 2; SIRT-3—sirtuin 3; SIRT-7—sirtuin 7. * p < 0.05 compared to control; *** p < 0.001 compared to control; ## p < 0.01 compared to 100 IS group; ### p < 0.001 compared to 100 IS group.

Among the studied SIRTs, only the expression of the SIRT-2 gene was significantly elevated in rats chronically exposed to both doses of IS (p < 0.001) compared to controls. In turn, SIRT-3 and SIRT-7 gene expressions were significantly lowered in 200 IS group compared to CON and 100 IS group (p < 0.001, and p < 0.01, respectively). We did not notice significant changes in SIRT-1 mRNA levels in both experimental groups (Fig. 3A–D).

The relations between expression of SIRTs, OATs and AhR gene pathway were shown in Table 3. In bone of rats exposed to 100 IS, the positive correlations were noticed between OAT-1, CYP1A2, SIRT-1 and SIRT-3. SIRT-1 gene was strongly associated with SIRT-3 and SIRT-7 expression, and the expression of SIRT-1 and SIRT-3 were strongly associated to AhRR gene expression. SIRT-1 also correlated with CYP1A2. In turn, SIRT-2 mRNA level was associated with AhR gene expression. Moreover, we observed a tendency to correlation between AhRR and SIRT-7 (R = 0.485, p = 0.057), as well as between SIRT-7 and SIRT-2 (R = 0.494, p = 0.050).

**Table 3:** The interrelationships between bone expression of SIRTs genes and AhR pathway genes in bone of rats after chronic exposure to IS.

	100 IS					200 IS			
	SIRT-1	SIRT-2	SIRT-3	SIRT-7		SIRT-1	SIRT-2	SIRT-3	SIRT-7
SIRT-1		R = 0.238 NS	**R = 0.691** **p = 0.003**	**R = 0.782** **p = 0.0003**	SIRT-1		*R* = *0.444* *p* = *0.084*	R = − 0.047 NS	*R* = *0.474* *p* = *0.064*
SIRT-2	R = 0.238 NS		R = 0.103 NS	R = 0.494 p = 0.050	SIRT-2	*R* = *0.444* *p* = *0.084*		*R* = *0.471* *p* = *0.066*	**R = 0.891** **p < 0.0001**
SIRT-3	**R = 0.691** **p = 0.003**	R = 0.103 NS		*R* = *0.429* *p* = *0.087*	SIRT-3	R = − 0.047 NS	*R* = *0.471* *p* = *0.066*		**R = 0.568** **p = 0.022**
OAT-1	*R* = *0.522* *p* = *0.067*	*R* = *0.516* *p* = *0.071*	**R = 0.571** **p = 0.041**	*R* = *0.500* *p* = *0.082*	OAT-1	*R* = *0.474* *p* = *0.064*	**R = 0.892** **p < 0.0001**	**R = 0.567** **p = 0.021**	**R = 0.991** **p < 0.0001**
AhR	R = 0.082 NS	**R = 0.529** **p = 0.035**	R = − 0.215 NS	R = 0.253 NS	AhR	**R = 0.725** **p = 0.002**	**R = 0.721** **p = 0.002**	R = 0.054 NS	**R = 0.625** **p = 0.013**
ARNT	R = 0.285 NS	R = 0.385 NS	R = − 0.018 NS	R = 0.206 NS	ARNT	R = 0.332 NS	**R = 0.765** **p = 0.0005**	R = 0.397 NS	**R = 0.729** **p = 0.001**
AhRR	**R = 0.635** **p = 0.008**	R = 0.368 NS	**R = 0.829** **p < 0.0001**	*R* = *0.485* *p* = *0.057*	AhRR	**R = 0.547** **p = 0.028**	**R = 0.503** **p = 0.047**	R = 0.194 NS	R = 0.421 NS
CYP1A2	**R = 0.506** **p = 0.045**	*R* = *0.447* *p* = *0.082*	*R* = *0.450* *p* = *0.080*	*R* = *0.479* *p* = *0.060*	CYP1A2	R = 0.403 NS	**R = 0.632** **p = 0.009**	**R = 0.706** **p = 0.002**	**R = 0.704** **p = 0.003**

AhR—aryl hydrocarbon receptor; AhRR—aryl hydrocarbon receptor repressor; ARNT—aryl hydrocarbon receptor nuclear translocator; 100 IS—group receiving indoxyl sulphate in the dose of 100 mg/kg b.w./day; 200 IS—group receiving indoxyl sulphate in the dose of 200 mg/kg b.w./day; OAT-1—organic anion transporter 1; SIRT-1—sirtuin 1; SIRT-2—sirtuin 2; SIRT-3—sirtuin 3; SIRT-7—sirtuin 7.

Bold values mean that correlation was statistically significant. Tendencies are in italic.

In bone of 200 IS rats, SIRT-2 gene expression was strongly associated with SIRT-7, and tendency to relationship existed between SIRT-2, SIRT-7 and remaining SIRTs. The gene expression of SIRTs, except SIRT-1, was associated with OAT-1 expression, and both SIRT-2 and SIRT-7 mRNA levels were strongly and positively associated with all genes belonging to AhR pathway (except for CYP1A1). Moreover, SIRT-1 gene was associated with AhR and AhRR mRNA levels, whereas SIRT-3 was related only to CYP1A2 expression (Table 3).

In CON group, we only found inverse association between SIRT-7 gene expression and CYP1A2 mRNA levels (R = − 0.541, p = 0.030).

### The effect of chronic exposure to exogenous IS on level of oxidative DNA damage in trabecular and cortical bone tissue

**Figure 4:**
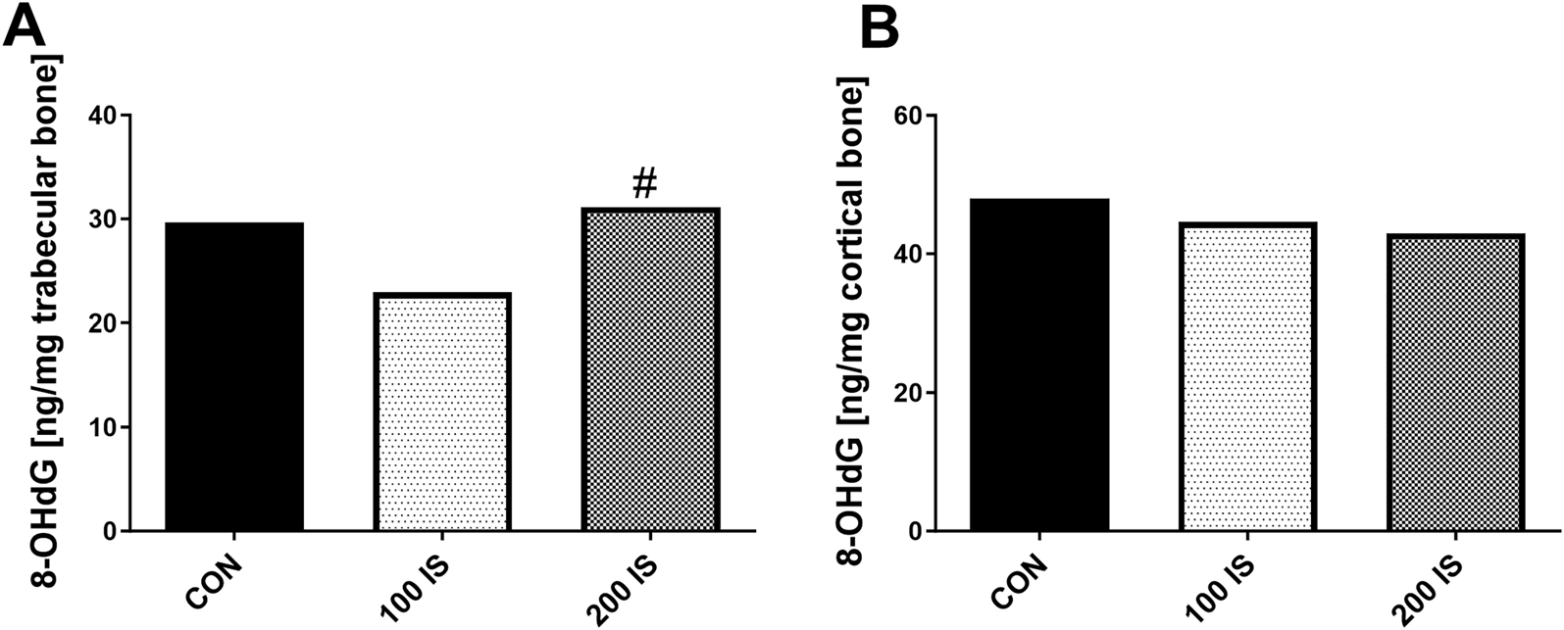
The level of 8-OHdG in trabecular (**A**) and cortical bone tissue (**B**) after chronic exposure to IS. Data are shown as the median. 8-OHdG—8-hydroxy-2'-deoxyguanosine; CON—control group; 100 IS—group receiving indoxyl sulfate in the dose of 100 mg/kg b.w./day; 200 IS—group receiving indoxyl sulfate in the dose of 200 mg/kg b.w./day; # p < 0.05 compared to 100 IS group.

Intracellular free ROS are produced as a result of normal metabolism, but their overproduction can impair DNA. The nonspecific DNA repair enzymes excise DNA lesions to release deoxynucleotides, which are enzymatically hydrolyzed to stable deoxynucleotides. Among numerous types of oxidative DNA damage, the formation of 8-hydroxy-2'-deoxyguanosine (8-OHdG) is a ubiquitous marker of oxidative DNA damage^27^. As shown in Fig. 4A, 8-OHdG level in trabecular bone tissue was lower in 100 IS group compared to 200 IS (p = 0.048), and it showed a downward trend compared to the control (p = 0.061). The median concentrations of 8-OHdG were 29.69 ng/mg (IQR 23.13–39.69) in CON group, 22.94 ng/mg (IQR 19.9–28.39) in 100 IS group, and 31.15 ng/mg (IQR 18.87–40.6) in 200 IS group. The results showed the absence of any differences between groups in cortical 8-OHdG levels (Fig. 4B). The median concentrations of 8-OHdG were 47.96 ng/mg (IQR 32.13–73.31) in CON group, 44.64 ng/mg (IQR 24.29–60.07) in 100 IS group, and 43.0 ng/mg (IQR 25.86–51.78) in 200 IS group.

In the group of rats administered with lower dose of IS the cortical 8-OHdG level was inversely associated with SIRT-1 and SIRT-7 gene expression, whereas 8-OHdG concentration in trabecular bone region positively correlated with CYP1A1 gene expression. Moreover, this oxidative DNA damage marker was inversely related to ARNT mRNA levels in trabecular bone region (Table 4, left side). In 200 IS rats, the inverse association was found between trabecular 8-OHdG levels and AhR, CYP1A2 gene expression, and the tendency to inverse correlation existed between 8-OHdG levels and SIRT-2 (R = − 0.495, p = 0.085), SIRT-7 gene expression (R = − 0.478, p = 0.098). In cortical part of bone, we noticed inverse relations between 8-OHdG levels and ARNT as well as tendency with SIRT-2 expression (R = − 0.516, p = 0.058).

**Table 4:** The relations between the level of 8-OHdG in trabecular and cortical bone tissue, and the expression of AhR pathway and sirtuins genes in bone of rats after chronic exposure to IS.

	100 IS		200 IS	
	Trabecular 8-OHdG	Cortical 8-OHdG	Trabecular 8-OHdG	Cortical 8-OHdG
AhR	R = − 0.121 NS	R = 0.150 NS	**R = **− **0.629** **p = 0.028**	R = − 0.390 NS
ARNT	**R = **− **0.676** **p = 0.011**	*R* = − *0.461* *p* = *0.083*	R = − 0.022 NS	**R = **− **0.542** **p = 0.044**
CYP1A1	**R = 0.599** **p = 0.031**	R = 0.129 NS	R = − 0.330 NS	R = 0.292 NS
CYP1A2	R = 0.253 NS	R = 0.067 NS	**R = **− **0.560** **p = 0.046**	R = − 0.051 NS
SIRT-1	R = − 0.412 NS	**R = **− **0.604** **p = 0.017**	R = − 0.247 NS	R = − 0.429 NS
SIRT-2	R = − 0.225 NS	R = 0.060 NS	*R* = − *0.495* *p* = *0.085*	*R* = − *0.516* *p* = *0.058*
SIRT-3	R = − 0.110 NS	*R* = − *0.461* *p* = *0.083*	R = − 0.088 NS	R = 0.120 NS
SIRT-7	*R* = − *0.489* *p* = *0.079*	**R = **− **0.514** **p = 0.049**	*R* = − *0.478* *p* = *0.098*	R = − 0.455 NS
Trabecular 8-OHdG		**R = 0.610** **p = 0.027**		R = 0.168 NS

8-OHdG—8-hydroxy-2'-deoxyguanosine; AhR—aryl hydrocarbon receptor; ARNT—aryl hydrocarbon receptor nuclear translocator; 100 IS—group receiving indoxyl sulphate in the dose of 100 mg/kg b.w./day; 200 IS—group receiving indoxyl sulphate in the dose of 200 mg/kg b.w./day; SIRT-1—sirtuin 1; SIRT-2—sirtuin 2; SIRT-3—sirtuin 3; SIRT-7—sirtuin 7.

Bold values mean that correlation was statistically significant. Tendencies are in italic.

### The effect of chronic exposure to exogenous IS on bone mineral status

**Figure 5:**
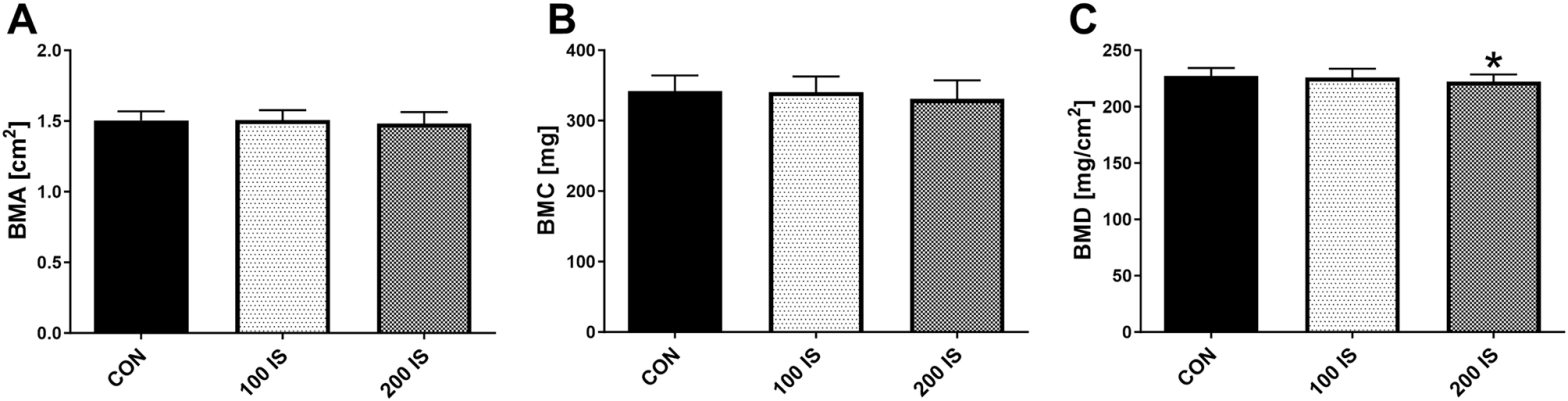
The bone mineral status of rats after chronic exposure to IS. Data are shown as the mean ± SD. BMA—bone mineral area; BMC—bone mineral content; BMD—bone mineral density; CON—control group; 100 IS—group receiving indoxyl sulfate in the dose of 100 mg/kg b.w./day; 200 IS—group receiving indoxyl sulfate in the dose of 200 mg/kg b.w./day; * p < 0.05 compared to control.

As shown in Fig. 5, there were no statistically significant differences between bone mineral area (BMA) [panel A] and bone mineral content (BMC) [panel B] between the all analyzed group. The values of bone mineral density (BMD) in 200 IS group were decreased compared to controls (p = 0.047) [panel C].

**Figure 6:**
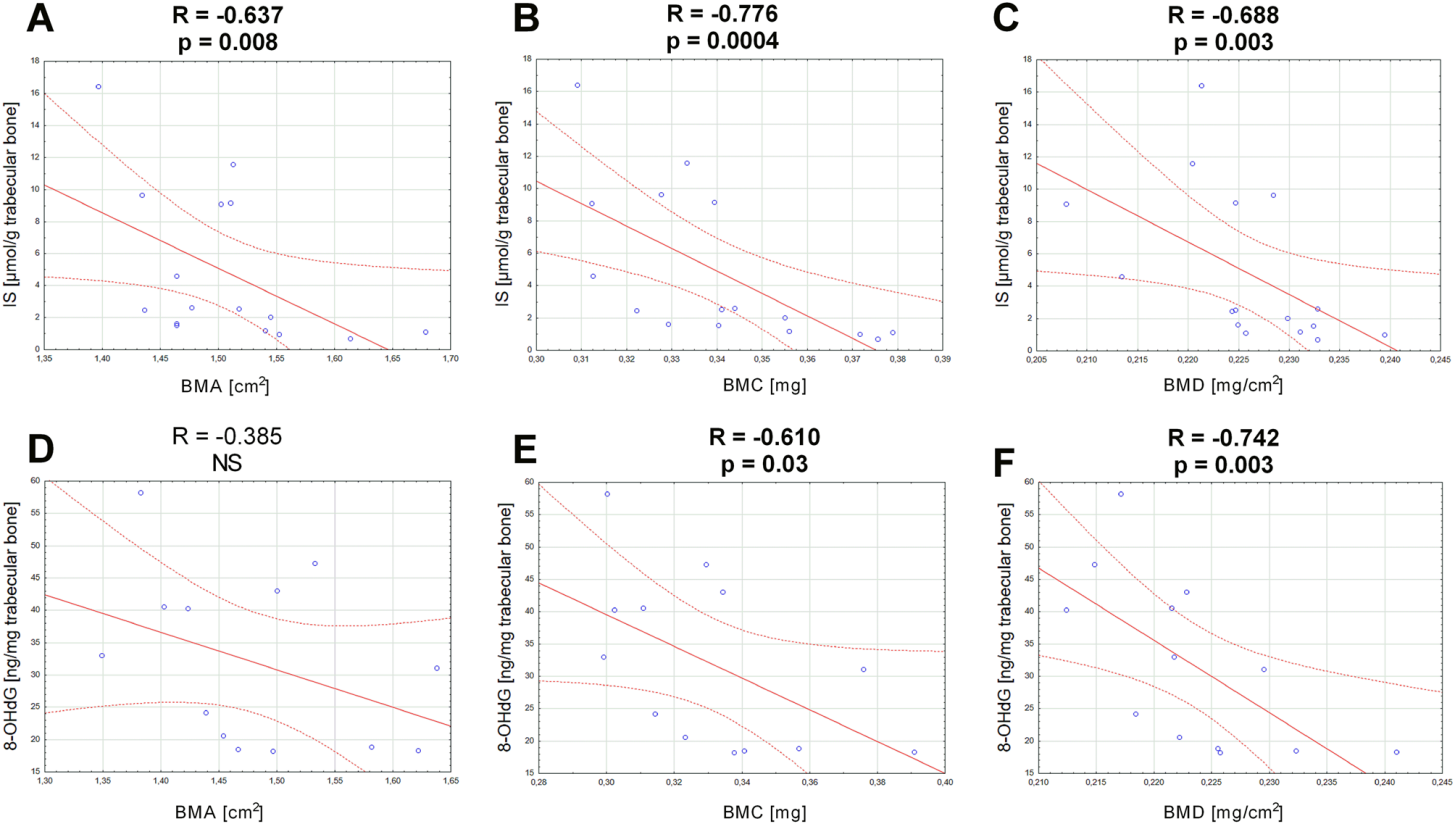
The effect of bone IS and bone oxidative status on mineral parameters of femurs in rats exposed to lower (**A**–**C**) and higher (**D**–**F**) dose of exogenous IS. 8-OHdG—8-hydroxy-2'-deoxyguanosine; BMA—bone mineral area; BMC—bone mineral content; BMD—bone mineral density; IS—indoxyl sulfate.

The analysis of relationships between bone mineral status and bone IS levels, activation of AhR pathway, SIRTs expression and bone oxidative status revealed that in the group of 100 IS the trabecular level of IS was strongly and inversely associated with BMA, BMC and BMD (Fig. 6, panels A–C). In contrary, in group of 200 IS, the parameters of bone mineral status, particularly BMC and BMD were inversely associated with trabecular oxidative status, representing by 8-OHdG levels (Fig. 6, panels D–F). There was no direct link between bone mineral status and SIRTs expression in both experimental groups, except of the weak association between SIRT-7 and bone mineral area (R = 0.490, p = 0.054) in 100 IS group.

## Discussion

Although several studies have provided evidence for the various harmful effects of IS on the body, its direct effect on bone homeostasis is not clear. To the best of our knowledge, this is the first study, in which we measured the levels of IS in in trabecular and cortical bone after chronic exposure of rats to IS. The plasma level of IS reached in this model, published in our previous study^24^, reflected IS levels observed previously by us and others in CKD patients^29,30^. This should make the possibility to reliably explore the effect of IS on the processes occurring in the bone tissue of CKD patients. Importantly, our model enables us to observe impact of IS solely, by eliminating the influence of other toxins creating uremic milieu. Using our model, we prevented interferences from other toxins, which is impossible in models of CKD that allows to observe the results of effects of many uremic retention solutes.

Previously, we showed the significantly increased plasma levels of IS in control group exposed to chronic ingestion of IS in the dose of 100 and 200 mg/kg b.w. for examination the effect of this uremic toxin on the hemostatic system and arterial thrombosis^24^. In the present study, we used the femurs of these rats to investigate the effect of chronic exposure to this uremic toxin on IS-mediated signaling in bone, and its significance for bone mineralization. We observed the accumulation of IS only in trabecular, more metabolically active bone tissue of these rats. Interestingly, the levels of IS in this bone region were not dependent on the dose used (Fig. 1A). Because OATs mediate IS uptake into bone cells^31,32^, we measured the expression of OAT-1 and -3, which are known to be transporters with high affinity to IS^27^ in bone of studied rats. Obtained results showed practically inhibition of both transporters in 200 IS group (Fig. 1C,D). Similarly to our results, the reduced renal mRNA and protein expression of OAT-1/3 was previously found in rat adenine-induced CKD model^33,34^, suggesting that rats with renal failure can be regarded as „OAT-1/3 down-regulation model”. However, the kidney function of our animals that received IS was only slightly reduced compared to controls^24^, so we think that the high circulating IS could be rather responsible for such significant OATs suppression in our model. This is in agreement with the previous study of Enomoto et al.^27^, who examined the interactions of IS with OATs, using proximal tubule cells stably expressing these transporters.

AhR is a cytoplasmic receptor of several exogenous and endogenous molecules that can control bone homeostasis. It has been shown that the exposure to dioxin, which is the most widely known exogenous AhR ligand, resulted in imbalance of bone remodeling and mechanically weaker bones^35^. CKD is characterized by the accumulation of a mixture of uremic toxins, and IS, which belongs to tryptophan metabolites, represents both important uremic toxin and potent AhR ligand^36,37^. In the present study, we measured the expression of AhR pathway genes in bone of rats after chronic exposure to IS (Fig. 2), and we analyzed the associations between their expression (Table 2). We observed the dose-dependent differences in the activation of AhR pathway. The exposure to IS in the lower dose (100 IS) strongly increased AhRR gene expression, but practically did not affect CYP1A1 mRNA level. This is in line with observation of Schanz et al.^38^ that cells that highly express the AhRR display only mild CYP1A1 expression. Typically, once induced AhRR competes with the AhR for dimerization with ARNT, and AhRR-ARNT complex binds XRE but does not initiate transcription of AhR-target genes^39^. Interestingly, we noticed the increased CYP1A2 gene expression, which was strongly AhRR-dependent. In contrast to CYP1A1, which is a readily inducible AhR target gene encoding an important xenobiotic metabolizing enzyme, CYP1A2 is usually expressed constitutively and metabolizes some endogenous substances^40^. Thus, the elevated mRNA level of CYP1A2 in rats exposed to 100 IS may indirectly indicate an active metabolism of IS, and therefore manifest the protective properties of CYP1A2 in this group.

The rats treated with higher dose of IS (200 IS) revealed the significant decrease in the expression of AhRR and both CYP1A genes, whereas the expression of AhR-ARNT complex tended to increase. In this group, AhR mRNA (but not AhRR) was directly associated with CYP1A2 gene expression, and AhR-ARNT-AhRR-CYP1A2 axis was positively linked with OAT-1 gene expression. These results suggest that higher dose chronically administered IS can disrupt the classical negative regulatory loop, in which AhR ligand could be rapidly degraded. As a consequence, the persistent overstimulation of AhR can be harmful for physiological processes in bone of these animals, as have been previously described by us and other authors^41,42^. Another interesting observation resulting from this study was that plasma and bone IS can diversely regulate CYP1A genes expression, as plasma IS levels were related to CYP1A1, whereas trabecular IS concentrations affected CYP1A2 gene expression in each of the studied group.

SIRTs are NAD + cofactor-dependent histone deacetylases (class III–HDAC), which take part in many processes regulating biological functions of cells, like cell cycle, cellular metabolism, mitochondrial function, protection against oxidative stress and inflammation^43,44^. SIRTs are present in many different organs including bones, but until now, little is known about the role of SIRTs in maintaining bone health^11,45^. We measured the expression of four SIRTs in bone of rats exposed to IS, namely SIRT-1, SIRT-2, SIRT-3 and SIRT-7. These SIRTs differ from each other in subcellular localization and function: SIRT-1 and SIRT-7 are nuclear SIRTs, and they are responsible for modulation of gene expression and DNA repair^46,47^, SIRT-2 is mostly found in the cytoplasm, and it plays an essential role in oxidative stress protection^13^, whereas SIRT-3 is located in the mitochondria and contribute to the regulation of ATP production, antioxidant defenses and energy metabolism^48^. However, there are data that SIRT-1, -2 and -3 can migrate between organelles^43^.

In 100 IS group we observed increased expression of SIRT-2, and slight but not significant elevation of the rest of SIRTs, which were related to CYP1A2 gene expression, and majority of them (except for SIRT-2) were also associated with AhRR mRNA levels. The exposure of animals to higher dose of IS resulting in diverse regulation of SIRTs expression. While SIRT-3 and SIRT-7 mRNA levels were significantly reduced in this group compared to 100 IS and controls, SIRT-1 expression remained constant, and SIRT-2 gradually increased (Fig. 3). Interestingly, the disrupted association between CYP1A2 and AhRR resulted in reduced expression of SIRT-3 and SIRT-7, whereas the direct relation between CYP1A2 and AhR augmented expression of SIRT-1, and particularly SIRT-2 gene.

In the available literature, there are no data so far on the regulation of SIRTs by IS-dependent components of the AhR pathway in bone. Nonetheless, the previous study of Szychowski et al.^20^ showed that exposure of mouse neurons to triclosan increased the expression of the SIRT-1 and SIRT-3 proteins in response to AhR stimulation. On the other hand, Diani-Moore et al.^21^ found that SIRT-1 activity in cultured hepatocytes of chicken embryos was reduced as a result of activation of the AhR receptor by dioxin. Similarly, the activation of AhR decreased SIRT-3 activity in transgenic mice with constitutively active AhR^22^. Koizumi et al.^23^ proved that IS-AhR pathway reduced NAD + content and SIRT-1 activity in human umbilical vein endothelial cells (HUVECs), inducing endothelial senescence. In our previous study, performed on these same animals, we show for the first time that chronic exposure to IS led to reduced aortic contents of SIRT-1 and SIRT-3^24^. Thus, the results of the present and previous studies indicated the existence of interaction between AhR and SIRTs, which seem to be depend on the type, concentration and duration of exposure to the used AhR agonist, as well as the subcellular localization of the SIRTs.

Recently, the abundant evidence shows that CKD is pro-oxidative state^49^ and the excessive generation of ROS are considered to be major mediators of numerous physiological complications in CKD patients, including CKD-MBD development^50^. Among the various metabolites that accumulate in the plasma of CKD patients, IS—a typical uremic solute, has been shown as an inducer of oxidative stress, modifying the balance between pro- and antioxidant mechanisms both in vitro condition^51–53^, and in patients with CKD^30^. Although most literature reports emphasize the pro-oxidative nature of IS, it has been also reported that IS can balance the oxidative stress in CKD, based on its physiological concentration in serum^54,55^. In the presence of concentrations of less than 10 µM in HUVECs, IS showed radical scavenging ability against superoxide generation in lipopolysaccharide-stimulated neutrophils^54^. It has been also shown that IS serves as an endogenous antioxidant to eliminate superoxides in the blood, protecting endothelial cells from oxidative damage under physiological conditions^55^.

To address whether AhR induction by IS at bone level may led to generation of oxidative stress, we measured 8-OHdG, which is recognized marker of oxidative DNA damage^28^ in homogenates from both bone regions. As has been presented on Fig. 4, the levels of 8-OHdG was decreased in trabecular bone tissue in 100 IS group compared to 200 IS group, and tended to be lower even in comparison with controls. The association observed between 8-OHdG and CYP1A1 (but not CYP1A2) in this group confirmed that CYP1A1 activation is responsible for ROS generation^56,57^. In contrast, the inverse association was observed between trabecular 8-OHdG concentration and AhR-CYP1A2 axis in rats received the higher dose of IS. These results suggest that in bone of animals exposed to lower dose of IS, the negative regulatory loop between ARNT-AhRR is able to protect the cells against CYP1A1-dependent ROS generation. On the other hand, a protective effect of CYP1A2 on ROS formation has been demonstrated^58^. In the current study, we observed the inverse associations between 8-OHdG levels and SIRTs expression, which were particularly seen in cortical bone (Table 4), where the increase in the oxidative DNA damage marker was not observed (Fig. 4B). As has been reviewed by Singh et al.^59^, SIRTs constitute an integral part of cellular defense against ROS formation, and each of them has a distinct subcellular localization, which allows rapidly sensing and responding to changes in subcellular ROS within the cellular organelles, like mitochondria, nucleus, and cytoplasm. In 100 IS group, we noticed the strong relations between nuclear SIRT-1 and SIRT-7, as well as between them and mitochondrial SIRT-3, suggesting that these SIRTs may interact closely to coregulate the levels of oxidative DNA damage In 200 IS group, the strong association was only seen between SIRT-2 and SIRT-7, and these SIRTs seem to be the major contributors of protection against oxidative DNA damage in both bone regions (Table 4, right side). Based on the above results, we created working hypothesis that after exposure to low dose of IS, the protective capacity of CYP1A2-SIRTs axis might exceed CYP1A1-dependent ROS production. However, after exposure to higher dose of IS, in the conditions of continual IS-mediated AhR activation, this protective mechanism can be disrupted, leading to increased oxidative DNA damage in trabecular bone region.

The last aim of the present study was to establish, if this specific intracellular target of IS could have the physiological relevance in relation to bone health in CKD. Osteoporosis and reduced bone mineral density (BMD) are the common complications related to uremia^60^. BMD measurements by Dual energy X-ray absorptiometry (DXA) are currently recommended for assessment of bone status in the CKD population by Kidney Disease Improving Global Outcomes (KDIGO) guidelines, as low BMD can predict fracture risk^61^. In the present study, the parameters of femoral mineral status, like bone mineral area (BMA) and bone mineral content (BMC) were unchanged in all studied groups, but BMD values of rats exposed to the higher dose of IS were reduced compared to healthy animals (Fig. 5). Interestingly, there was no clear, direct association between the parameters of bone mineral status and SIRTs, as well as AhR pathway gene expression in rats receiving IS. However, the strong, negative impact of IS content in trabecular bone was noticed in relation to BMA, BMC and BMD values in 100 IS group (Fig. 6A–C). In rats exposed to IS at the dose of 200 mg/kg b.w., the level of oxidative DNA damage was inversely associated with these parameters, especially with BMD (Fig. 6D–F). These results showed that even a little content of IS in bone tissue, through activation of AhR system in situ*,* is able unfavorable affecting bone mineral status. Moreover, these results confirmed our hypothesis that protective effect of AhR-dependent induction of SIRTs expression after exposure to low dose of IS can effectively balance oxidative DNA damage, counteracting BMD reduction.

## Conclusions

**Figure 7:**
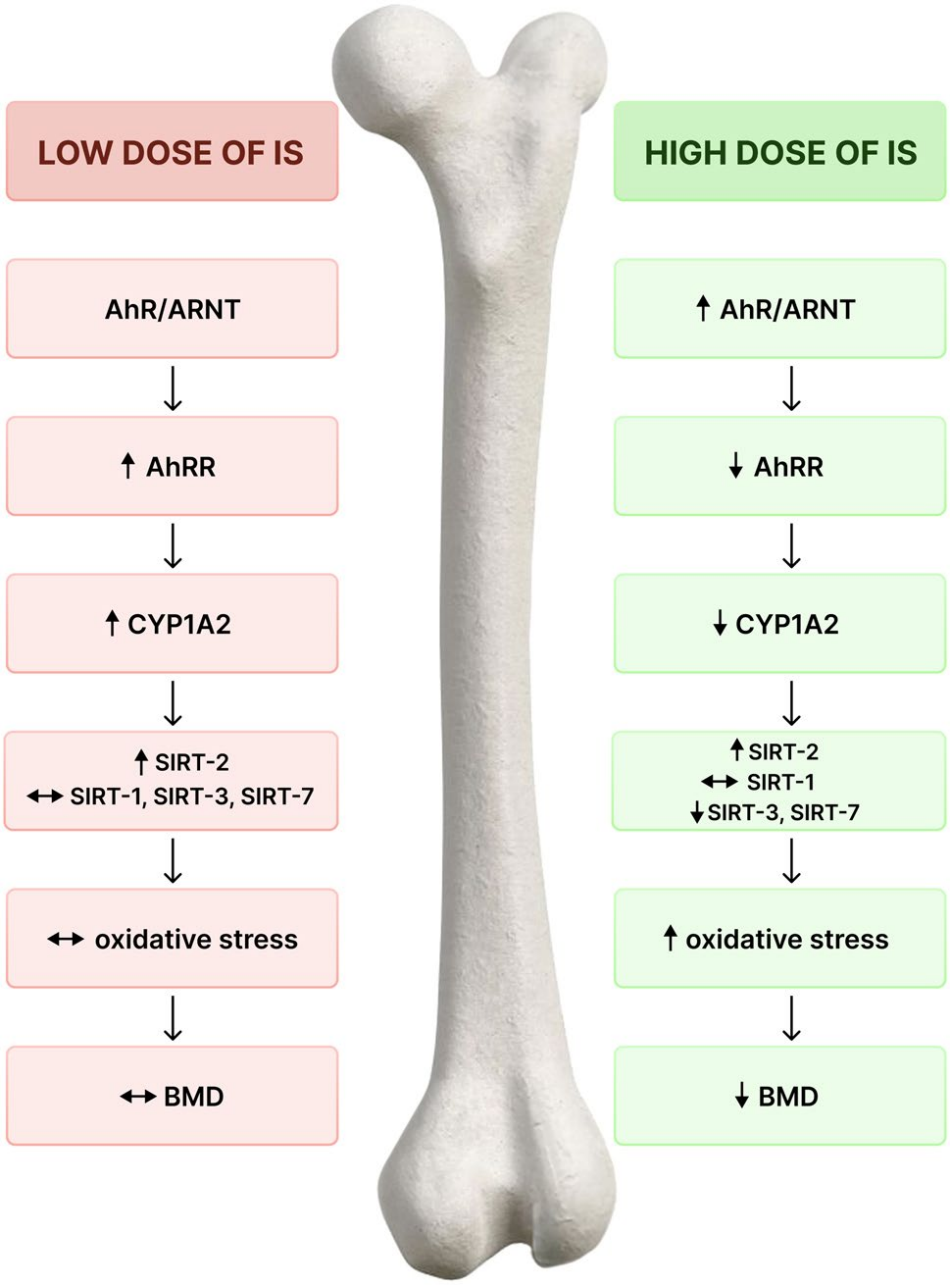
The proposed mechanism of both protective (left side) as well as detrimental (right side) action of IS in CKD-related osteoporosis. AhR—aryl hydrocarbon receptor; AhRR—aryl hydrocarbon receptor repressor; ARNT—aryl hydrocarbon receptor nuclear translocator; BMD—bone mineral density; SIRT-1—sirtuin 1; SIRT-2—sirtuin 2; SIRT-3—sirtuin 3; SIRT-7—sirtuin 7. Legend: The low dose of IS, trough the activation of AhR/AhRR/CYP1A2 axis and SIRTs expression, can effectively balance the oxidative DNA damage in bone tissue, maintaining proper bone mineral status. At the high dose of IS this protective mechanism is interrupted, resulting in increase of bone oxidative DNA damage that leads to reduced BMD.

Recently, some studies suggested that IS and other uremic toxins may contribute to the loss of bone quantity and quality in CKD^36^ but majority of these works were conducted in bone cell culture models with high micromolar IS concentration, or in animal CKD models, so their physiological relevance remain uncertain. In contrast, our data clearly demonstrate that chronic exposure of control group to IS can modulate AhR-depending biological signaling at bone level, affecting SIRTs expression, oxidative DNA damage level and bone mineral status. The results presented here describe for the first time a specific, dose-dependent intracellular target of IS in bone, on the basis of which we propose the mechanism (Fig. 7), showing both protective, as well as detrimental nature of IS in CKD-related osteoporosis. Because the patients with CKD are continuously exposed to high IS levels^30^, it would be expected that their bone AhR should be fully and sustained activated, that might predispose these patients to bone loss and risk of fracture.

## Data Availability

The datasets used and/or analyzed during the current study are available from the corresponding author on reasonable request.
